# A Guide to Brighter Phosphors‐Linking Luminescence Properties to Doping Homogeneity Probed by NMR

**DOI:** 10.1002/cphc.201900790

**Published:** 2019-11-07

**Authors:** Wenyu Li, Matthias Adlung, Qianyun Zhang, Claudia Wickleder, Jörn Schmedt auf der Günne

**Affiliations:** ^1^ Department of Chemistry and Biology Chemistry University of Siegen Adolf-Reichwein-Str. 2 57076 Siegen Germany; ^2^ Department of Chemistry and Biology Chemistry University of Siegen Adolf-Reichwein-Str. 2 57076 Siegen Germany

**Keywords:** doping homogeneity, lifetime, luminescence, NMR, XRD

## Abstract

Crystalline powders of Ln^3+^ doped LaPO_4_ (Ln=Nd, Gd, Dy, Ho, Er, Tm, Yb) have been synthesized to serve in a case study for linking doping homogeneity as determined by NMR to luminescent properties. Samples obtained via different synthesis methods act as examples of homo‐ and inhomogeneous doping. The sample quality was verified by X‐ray diffraction. The homogeneously doped samples show improved luminescent properties in terms of brightness and lifetime which is consistent with the interpretation that, NMR visibility curves probe the distribution of paramagnetic dopants on a similar length scale as necessary for an efficient energy transfer in crystalline phosphors i. e. between sensitizers and activators, and to killer sites. Thus “NMR homogeneity” as observed by visibility curves may serve as a tool to optimize luminescent materials.

## Graphical abstract

**NMR allows distinguishing** homogeneous from heterogeneous doping situations, which makes phosphors convert light more efficiently.

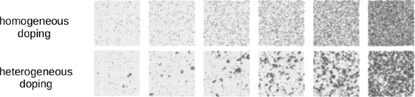

## 1 Introduction

**Figure 1:**
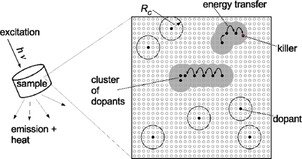
A schematic sketch describing how the dopant distribution affects the luminescence behavior. The crystalline host is shown by its potential doping sites (small empty circles) and the sites filled with dopants (filled black circles). Dopants can be activators (A) and/or sensitizers (S) depending on the system. Energy transfer may happen between either of them, i. e. S to A (SA) or S to S (SS) if they are closer than the critical distance^16^ *R*_C_ (big circles around a dopant), which differs for different dopants and different transfer processes. For mono‐doped systems, concentration quenching^6^ of an emission band may be ascribed to energy migration of the excited state to a cluster site leading to cross‐relaxation or to a killer site (red circle) leading to non‐radiative conversion to the ground state.

**Figure 2:**
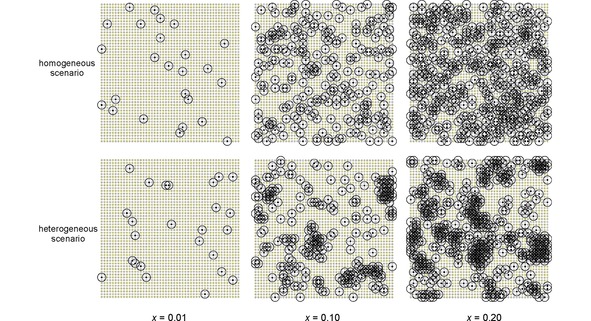
A simplified two‐dimensional illustration of a homogeneous (top row) and a heterogeneous (bottom row) doping scenario in a crystalline host where a certain fraction *x* of dopable sites (small circles) is filled with paramagnetic dopants (black filled circles). In terms of NMR visibility only signals from the volume outside the blind spheres^26^ (big circles with *r*_0_) can be picked up. In terms of luminescence the radii of these spheres (big circles) could be interpreted as the critical energy transfer distance^16^ *R*_c_. The radii *r*_0_ and *R*_c_ are not the same but typical values fall into a similar range: Å – few nm.^17^, ^18^, ^19^, ^20^, ^26^, ^35^, ^36^

Paramagnetic dopants, especially the paramagnetic lanthanide ions, play an important role in various applications, for example Y_3_Al_5_O_12_:Ce^3+^ is used as scintillator material,^1^ Y_2_O_3_ : Eu^3+^ in cathode ray tubes,^2^ Gd_2_O_2_S : Tb^3+^ as X‐ray phosphor,^3^ SrAl_2_O_4_ : Eu^2+^,Dy^3+^ as long persistent phosphor^4^ and Y_3_Al_5_O_12_ : Nd^3+^ in solid‐state lasers.^5^ In case of the application scenario of light‐converting phosphors, brightness, efficiency and lifetime are related to the local pair distance^6^ and the effective dopant concentration,^7^ which are both microscopically related to the dopant distribution. Especially for brightness, i. e. quantum yields, phosphors may suffer from concentration quenching^6^, ^8^ at high doping level which inhibits higher emission intensities. A homogeneous distribution of dopants ensures a high effective doping concentration while it avoids concentration quenching^6^ at low doping concentration and therefore improves quantum yields (Figure 1 and 2).

Distribution of dopants can be investigated by different techniques, for example by X‐ray diffraction (XRD),^9^, ^10^ X‐ray photoelectron spectroscopy (XPS)^10^ or energy dispersive X‐ray spectroscopy (EDX).^11^ In general, the concept “homogeneity” as defined by IUPAC^12^ is related to a defined quantity of a material, i. e. volume or length scale: optical glasses depict long‐range homogeneity when investigated by optical microscopic techniques (visible light), but may show inhomogeneity (heterogeneity) on an atomic scale (electron microscopy).^13^ Here the term “homogeneous doping” is used to refer to a random, i.e. statistical, substitutional doping of a crystalline host material (Figure 2). Depending on the analytical technique and required length scale, a sample could thus appear to be homogeneous and inhomogeneous at the same time. Besides, some techniques like XPS and EDX are more surface sensitive or have a smaller analyzed volume,^14^ while others sense bulk properties^15^ as for example laboratory powder XRD. Different analytical techniques often nicely complement one another with respect to length scale and surface sensitivity. However, analysis of homogeneity on an atomic scale of dopants added in the low‐percent range turns out to be a non‐trivial problem.

The role of energy transfer processes,^6^, ^16^, ^17^, ^18^ which may happen between sensitizers or between sensitizer and activator, and from sensitizer to killer sites, can't be underestimated (Figure 1) for a better understanding of how the dopant distribution relates to luminescence performance. The processes operate over a distance called critical energy transfer distance *R_c_* which ranges from a few ångströms to a few nanometers.^17^, ^18^, ^19^, ^20^

Solid state NMR has been reported to be helpful for studying paramagnetic systems.^21^, ^22^, ^23^, ^24^, ^25^, ^26^ Especially, the distribution of paramagnetic dopants can be investigated by the spin‐lattice relaxation time,^27^, ^28^, ^29^, ^30^ hyperfine shifts^31^, ^32^ and the line‐broadening effect.^27^, ^33^, ^34^ Homogeneous distributions of Tb^3+^ and Eu^3+^ were shown to be correlated to NMR line broadening and positively related to the brightness of phosphors.^33^, ^34^

**Figure 3:**
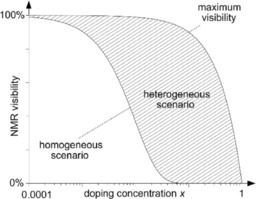
Typical NMR visibility curves *f*(*x*) as a function of the paramagnetic dopant concentration *x*. The NMR visibility is defined as the visible signal of the doped material normalized by the signal of the diamagnetic host.^35^ For a homogeneous doping scenario, the NMR signals vanish in the dead‐time of the spectrometer^36^ more efficiently thus *f*(*x*) approaches zero earlier as *x* increases. The visibility of the heterogeneous case *f*(*x*) is located in the dashed regime between the maximum visibility *f*_max_(*x*) (upper solid line) and the visibility of a homogeneous doping scenario. The NMR visibility is always smaller for a homogeneous (lower solid line) than for a heterogeneous doping scenario (compare Figure 2) at the same doping concentration.

A disadvantage of a lineshape analysis is that it implicitly only refers to the NMR visible part of the compound but not to the nuclei inside the blind sphere, i. e. in direct vicinity of paramagnetic centers, for which the signal may vanish within the dead‐time of spectrometer. An alternative to the lineshape analysis approach is the visibility function, i. e. the observed peak area as a function of doping concentration which was also shown to be related to doping homogeneity (Figure 3).^35^ It is interesting to note that for lanthanide ions the blind sphere radii^26^, ^35^, ^36^ and the critical energy transfer distances^17^, ^18^, ^19^, ^20^ share a similar size range. The aim of this contribution is to relate NMR doping homogeneity to the luminescence properties of inorganic phosphors on the basis of the NMR visibility function^35^ for the first time. For this case study LaPO_4_ was chosen as a diamagnetic inorganic host and different Ln^3+^ ions (Ln=Nd, Gd, Dy, Ho, Er, Tm, Yb) served as paramagnetic dopants which allows to determine blind sphere radii by ^31^P NMR and sample quality by XRD at the same time. Besides LaPO_4_:Ln(III) is a reasonable phosphor^37^, ^38^, ^39^ so that luminescence properties (lifetime and brightness) could be determined. If the working hypothesis is true, that the NMR length scale given by the blind sphere radius is similar to the distance relevant to energy transfer in lanthanide doped phosphors, then it should be possible to observe differences in luminescence properties between homogeneously and inhomogeneously doped samples.

## 2 Results and Discussion

### 2.1 XRD Homogeneity

In a first step two sample series were obtained by different synthesis routes. Their doping homogeneity was evaluated by XRD, i. e. by Rietveld refinement and Vegard's law.^40^ The corresponding refinement results (Supporting information Figure S1–S4) of the La_1‐*x*_Ln_*x*_PO_4_ series (Ln=Nd, Dy, Ho, Yb), which were obtained by **co‐precipitation**, show a linear correlation of the lattice parameters with the doping concentration *x*. Such fulfillment of Vegard's law is often obeyed by **homogeneously** doped samples which follow random substitutional replacement of ions.^41^

For the samples obtained via a **solid‐state reaction** a phase separation of LnPO_4_ and LaPO_4_ becomes evident at high doping concentration (*x*≥0.2) from the powder diffractograms (Supporting Information Figure S5). Phase separation is a typical case of **heterogeneity**. Note that XRD requires the samples to be doped to a high degree (herein about *x*≥0.05) to obtain lattice parameter changes which are significant, while doping levels relevant to luminescence properties often require low lanthanide doping concentrations, for instance *x* <5 %.

### 2.2 NMR Homogeneity

In a second stage these sample were investigated by quantitative ^31^P NMR to test whether the NMR homogeneity agrees with XRD results at low doping concentration. Only one ^31^P NMR signal at around −4.6 ppm, which corresponds to monazite LaPO_4_, was observed for the La_1‐*x*_Ln_*x*_PO_4_ (Ln=Nd, Gd, Dy, Ho, Er, Tm, Yb). As the doping level *x* increases, the signal broadens without significant shifts and the peak area decreases (^31^P MAS NMR spectra stack plots in Supporting Information Figure S6–S11). Thus the situation in the investigated cases is simpler than in the cases of La_1‐*x*_Sm_*x*_PO_4_ 36 and La_1‐*x*_Eu_*x*_PO_4_,^32^ where different ^31^P signals could be observed.

**Figure 4:**
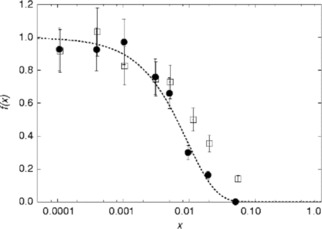
The normalized NMR visibility *f*(*x*) as a function of doping concentration *x*, for La_1‐*x*_Dy_*x*_PO_4_ obtained by the co‐precipitation method (circles) and by the solid‐state reaction method (squares). The dashed lines feature a fitted visibility function^27^ *f*(*x*)=exp(‐*ar*_0_^3^ *x*) with *a*=0.055/Å^3^ and *r*_0_=12.5 Å.

The resulting peak areas from one pulse experiments were calculated and tested by the visibility function.^35^, ^36^ Deviations from the theoretical visibility function for homogeneous doping indicate a lower degree of NMR homogeneity. By this comparison for seven Ln^3+^ dopants (Figure 4 for Dy^3+^ and for other Ln^3+^ ions (Supporting Information Figure S6–S11)) it is possible to conclude that the samples series obtained via a solid‐state‐reaction showed a lower degree of NMR homogeneity as compared to the co‐precipitated samples of the same dopant series. Therefore, differences in homogeneity can be traced via the NMR visibility function in the low‐doping regime. The result is in excellent agreement with the XRD analysis.

The homogeneity length scale of the NMR experiment is related to the radius of the blind sphere of the paramagnetic dopant. For Nd, Gd, Dy, Er, Ho, Tm, Yb in La_1‐*x*_Ln_*x*_PO_4_ the blind sphere radii are 5.5 Å, 13.5 Å, 12.5 Å, 10.5 Å, 10 Å, 9 Å and 5.8 Å respectively.^36^ Based on the differences in homogeneity traced via the NMR visibility curves (Figure 4 and Figure S6–S11), it may be concluded that only the co‐precipitated samples qualify as homogeneously doped on a length scale of about 1 nm.

### 2.3 Luminescence Spectra

What remains to be shown is that homogeneity on the nm‐scale correlates with luminescence properties. From the different available doping series only Dy^3+^ doped LaPO_4_ was chosen for luminescence measurements. Quantitative excitation and emission spectra for the Dy^3+^ doped samples with *x*=0.05 (Figure 4) show their most intense emissions bands at 477 and 572 nm which can be assigned to transitions^42^, ^43^ ^4^F_9/2_→^6^H_15/2_ and ^4^F_9/2_→^6^H_13/2_, respectively.

**Figure 5:**
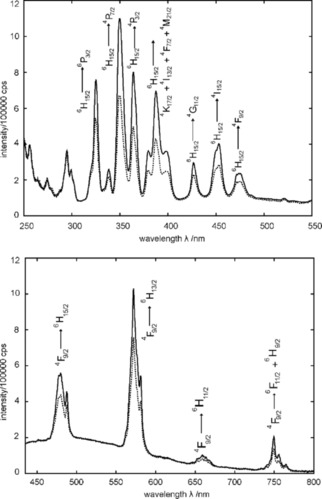
Excitation (top) and emission (bottom) spectra of La_0.95_Dy_0.05_PO_4_ obtained by co‐precipitation (solid line) and solid‐state reaction (dashed line). The excitation spectra were recorded at an emission wavelength of λ_em_=572 nm. The emission spectra were measured at an excitation wavelength of λ_ex_=325 nm.

An increase in brightness can be observed (Figure 5) for the La_0.95_Dy_0.05_PO_4_ samples which feature a more homogeneous dopant distribution according to the NMR and XRD analysis.

### 2.4 Luminescence Lifetimes

To obtain independent evidence of doping homogeneity lifetime measurements of the two sample series of La_1‐*x*_Dy_*x*_PO_4_ were obtained (lifetime curves: Supporting Information Figure S12–S18). Qualitatively the individual measurements show a transition from mono‐exponentially decaying lifetime functions to a complex behavior with increasing doping concentration *x*.

How can this behavior qualitatively be understood and what can be learned about the distribution of dopants from lifetime measurements?

The lifetime functions applying to isolated ions are well established, describe the intensity decay as a mono‐exponential function and require excitation and emission to occur on the same ion S.^16^, ^18^

$$I\left(t\right)=I_{0}\exp\left(-\frac{t}{\tau_{0}}\right)$$

The same model also applies when the energy is transferred from one S atom to another one (SS transfer) between excitation and emission.

When a photon is excited on an S atom (sensitizer) transferred to an activator (A) or a killer site (SA transfer),^18^ deviations from mono‐exponential functions are expected.^16^, ^18^

$$I\left(t\right)=I_{0}\exp\left(-\frac{t}{\tau_{0}}-C\;t^{\frac{3}{n}}\right)$$

*C* is a constant that relates to both concentration of A, and the interaction strength between S and A, while *n* depends on the electric multipole interaction (*n*=6, 8 or 10 for dipole‐dipole, dipole‐quadrupole or quadrupole‐quadrupole interaction, respectively).^44^ If SA and SS transfers are combined complicated non‐monoexponential models apply.^18^ Note that in a doping series with variable doping concentration *x* the lifetime of the activators will reflect their individual environments, i. e. clustered activators which are subject to cross‐relaxation and isolated activators will exist side by side and their lifetime curves can best be described as a sum of monoexponential curves with different lifetime values. Lifetime reduction by cross‐relaxation processes^16^, ^18^, ^42^ between Dy^3+^ ion pairs, for example (^4^F_9/2_, ^6^H_15/2_)→(^6^H_5/2_, ^6^F_7/2_) and (^4^F_9/2_, ^6^H_9/2_ (^6^F_11/2_))→(^6^H_15/2_, ^6^F_3/2_) is well estabilshed.^42^, ^43^

As shown in detail in the supporting information (Figure S12–S18) the lifetime curves of both the homogeneous and the heterogeneous scenario can be described using the same lifetimes $\tau_{1}$ , $\tau_{2}$ and $\tau_{3}$ in the a tri‐exponential model (see Supporting Information for details).

$$I\left(x\right)=I_{0}\cdot \left(a_{1}\cdot e^{-\frac{x}{\tau_{1}}}+\;a_{2}\cdot e^{-\frac{x}{\tau_{2}}}\;{+\;a}_{3}\cdot e^{-\frac{x}{\tau_{3}}}\right)+I_{\mathrm{offset}}$$

$$1=a_{1}+a_{2}+a_{3}$$

$$\tau_{1}>\tau_{2}>\tau_{3}$$

The corresponding parameters could be extracted via synchronous least‐square fitting of all lifetime curves. This approach minimizes the number of fitting parameters and delivers a stable fitting model. The longest lifetime $\tau_{1}$ clearly can be assigned to the lifetime of isolated Dy ions, while the other two can be considered as fitting parameters to reflect different processes leading to shorter lifetimes or more complex decay functions in general. The parameters *a*_1_, *a*_2_ and *a*_3_ depend on the doping concentration and are the weights of the individual exponential curves, while *I*_0_ and *I*_offset_ depend on the individual measurements, i. e. the amount of experimental time spent on each experiment and background noise, respectively.

Lifetime measurements can be described both for the heterogeneous and homogeneous sample series in the low‐doping regime with the same lifetimes. Thus it is reasonable to argue that killer sites caused by different lattice defects, e. g. color centers, which should be synthesis dependent, have a negligible influence on the lifetime curves in this case. Impurities by other rare earth elements were neglected because of the used reagent purities. Thus the shortening of the lifetimes will be discussed based on the assumption that cross‐relaxation caused by cluster formation of Dy‐ions is the main mechanism for lifetime reduction.

**Figure 6:**
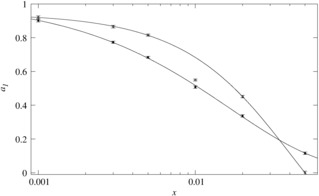
Weight *a*_1_ with error bars of the slowest decay process in a lifetime measurement obtained by a synchronous tri‐exponential fit of all lifetime curves at different doping concentrations *x*, for two La_1‐*x*_Dy_*x*_PO_4_ doping series: samples obtained by co‐precipitation (open circles), as an example for homogeneous doping and samples obtained via a solid‐state reaction (filled squares), as an example for heterogeneous doping according to NMR and XRD. The lines serve as a guide to the eye (homogeneous case (solid line) *a*_1_(*x*)=1.294 ⋅ exp(−(25.5 ⋅ *x*)^1.095^)–0.352, heterogeneous case *a*_1_(*x*)=1.018 ⋅ exp(−(60.4 ⋅ *x*)^0.729^+0.008). The fairly small statistical errors obtained via an error analysis indicate that the fit is stable and sample‐preparation dependent errors dominate.

How should the dopant distribution influence the weights *a_n_* as a function of doping concentration *x*? The weight *a*_1_ is a measure for the frequency of isolated dopants and is well defined because it describes the degree of mono‐exponentiality of the lifetime curves. The relative amount of isolated dopants (∼*a*_1_) decreases as the doping concentration *x* increases in both the homogeneous and the heterogeneous doping model (Figure 6).

The visualization (Figure 2) may help grasping the differences between the homogeneous and the heterogeneous doping scenario. In the low‐doping regime both the homogeneous and the heterogeneous case look similar (Figure 2), which reflects the stabilization of defects for entropic reasons and the formation of a thermodynamically stable solid solution of La_1‐*x*_Dy_*x*_PO_4_. Higher doping concentrations may cause demixing as the XRD measurements showed (Supporting Information Figure S5). The precise distribution of the dopants in the host depends on the preparation and the type of segregation process, for example spinodal demixing versus crystallization and growth, and Oswald ripening. The formation of clusters may be considered as a consequence of demixing and phase segregation. Note, that in the homogeneous doping scenario clusters will form simply for statistical reasons, especially in the high doping regime. In the low doping regime the frequency of isolated dopants, corresponding to the non‐overlapping spheres (Figure 2) with a radius of the critical energy transfer distance *R*_c_, would be higher in the homogeneous than in the heterogeneous case. However in the high doping regime the frequency of isolated dopants are expected to be higher in the heterogeneous than in the homogeneous scenario, because demixing generates islands of low‐doping between clusters of dopants in the heterogeneous case (Figure 2).

Following this argument the frequency of isolated dopants, i. e. weight *a*_1_, is then expected to start off with similar values for the homogeneous and heterogeneous doping scenario at low doping concentration (Figure 6). With increasing doping concentration, clusters would form which indirectly reduce the frequency of isolated dopants. Because clusters form more easily in the heterogeneous doping scenario its frequency of isolated dopants would be reduced faster. However the opposite behavior is expected for the even higher doping concentrations: while in the homogeneous case for statistical reasons almost all dopants would be part of a cluster (Figure 2), in the heterogeneous case a limited frequency of dopants could still remain in the space between the clusters. Consequently the frequency of isolated dopants as a function of doping concentration should feature a cross‐over for the homogeneous and the heterogeneous scenario. As can be seen in Figure 6, the expected behavior is observed which is an independent confirmation of the hypothesis that NMR homogeneity can be related to the luminescence properties, because blind sphere radii and critical distances in luminescence are of the same order of magnitude.

## 3 Conclusions

The main target of this contribution was to test whether homogeneity evaluated by the peak areas in solid state NMR shows consistency with the actual luminescence performance. To this end seven different sample series of lanthanide doped monazite LaPO_4_ were prepared. Sample homogeneity could be tested by XRD in this case because the doping concentration could be varied from *x* equals 0 to 100 %. NMR was able to provide the same information on the basis of the NMR visibility function, with the advantage that the visibility function does not require the samples to be doped up to 100 % but to much lower values. The established homogeneity of the samples was shown to be related to both fluorescence intensity and the lifetime of the excited states. The length scale of the NMR homogeneity criterion can be estimated as the blind‐sphere radius which takes values up to 1 nm approximately for Ln(III) dopants. The method is not restricted by the choice of the host structure as long as NMR nuclei are present. An application of this method to for example Ln(II) or Ln(III) doped halogenides, phosphates, borates or nitrides should be straight forward. Synthesis methods that produce samples with higher NMR homogeneity avoid the unnecessary consumption of dopant reagents. The activator ions can be used more efficiently and a higher light yield should be achievable. We conclude solid state NMR may act as a tool for the evaluation of different synthesis methods and for optimizing luminescence properties.

## Experimental Section

Two commonly used synthesis routines have been selected in order to create different degrees of doping homogeneity.

The co‐precipitation method: Ln_2_O_3_ (Ln=Nd, Gd, Dy, Er, Ho, Tm, Yb. Nd_2_O_3_ was bought from ChemPur, the rest from smart elements®. The purity is 99.999 % for Dy_2_O_3_ and 99.99 % for the rest) and La_2_O_3_ (Chempur, 99.99 %) were dissolved in concentrated nitric acid and later on mixed with excess NH_4_H_2_PO_4_ (VWR chemicals) solution. The resulting precipitates were centrifuged and washed with water and ethanol. The washed precipitates were dried at 80 °C overnight and sintered in corundum crucibles at 1000 °C for 4 h.

The “solid‐state reaction” method: stoichiometric amounts of Ln_2_O_3_, La_2_O_3_ and NH_4_H_2_PO_4_ were ground in an agate mortar and sintered in corundum crucibles at 1000 °C for 4 h.

Powder XRD measurements were performed on a Huber G621 diffractometer with Cu *K*_α1_ radiation (λ=0.15405931 nm) and Guinier camera in transmission geometry. Diffractograms were extracted from the image files, which were obtained by scanning photostimulable BaBrF : Eu^2+^ films with an image plate detector (Typhoon FLA 7000, λ=650 nm), by a home‐written program (“ipreader”, version 0.9).

The solid state NMR measurements were performed on a Bruker Avance II spectrometer at 7.05 T. Magic angle spinning (MAS) was done with 4 mm pencil rotors at 10 kHz spinning frequency with completely filled rotors with a home‐built McKay probe head. The spectra were acquired by direct excitation, with a dead time of 15 μs, and 90° pulses with a pulse length of typically 4–5 μs and repetition delays being longer than 5 times the *T*_1_ relaxation time to ensure quantitative measurements. In addition, quantification was assisted by a micro‐balance (Sartorius MC5). The deconvolution of peaks was performed by the program deconv2Dxy^45^ (version 0.4). Because an external referencing method was used for quantification we estimated that this scheme causes a relative error of the individual measurements of about 10 % being related to small changes in tuning, matching and in dielectric loss. The NMR visibility was calculated as observed peak area per mole of doped sample normalized by that of the non‐doped sample. The NMR visibility fitting function^35^ for homogeneously doped sample was shown to be $f\left(x\right)=exp\left(-{ar}_{0}^{3}x\right)$ , with *a*=4π*N*_hostUC_/3*V*_UC_=0.055/Å^3^ for monazite^46^ LaPO_4_, where *N*_hostUC_ is the number of “dopable” sites in the unit cell and *V*_UC_ is the volume of the unit cell. Herein *N*_hostUC_=4 and *V*_UC_=305.73 Å^3^. The variable *r*_0_ is the blind sphere radius of a paramagnetic ion.

Quantitative excitation and emission spectra were recorded on a FluoroMax HORIBA fluorescence spectrometer within a spectral range of 250–800 nm with an integrating sphere. The emission spectra were corrected for the sensitivity of the photomultiplier and the reflectivity of the integrating sphere. Decay times were measured at room temperature using a 75 W Xe flash attached to a Fluorolog 3 spectrometer (FL3‐22, Horiba). At both spectrometers the emission was detected by a photomultiplier R928P from Hamamatsu.

## Conflict of interest

The authors declare no conflict of interest.

## Supporting information

As a service to our authors and readers, this journal provides supporting information supplied by the authors. Such materials are peer reviewed and may be re‐organized for online delivery, but are not copy‐edited or typeset. Technical support issues arising from supporting information (other than missing files) should be addressed to the authors.

Supplementary
