# Supplementary material for: A Guide to Brighter Phosphors‐Linking Luminescence Properties to Doping Homogeneity Probed by NMR
Source: Chemphyschem. 2019 Nov 7;20(23):3245–50. doi: 10.1002/cphc.201900790 (PMC6916297; doi:10.1002/cphc.201900790)
Supplement: Supplementary file 1 — Supplementary [file CPHC-20-3245-s001.pdf]

CHEMPHYSCHEM

Supporting Information

© Copyright Wiley-VCH Verlag GmbH & Co. KGaA, 69451 Weinheim, 2019

A Guide to Brighter Phosphors-Linking Luminescence Properties to Doping Homogeneity Probed by NMR

Wenyu Li, Matthias Adlung, Qianyun Zhang, Claudia Wickleder, and
Jörn Schmedt auf der Günne*© 2019 The Authors. Published by Wiley-VCH Verlag GmbH & Co. KGaA.
This is an open access article under the terms of the Creative Commons Attribution License, which permits use, distribution and reproduction in any medium, provided the original work is properly cited.

A guide to brighter phosphors - linking luminescence properties to doping homogeneity probed by NMR

Wenyu Li[a], Matthias Adlung[b], Qianyun Zhang[a], Claudia Wickleder[b], Jörn Schmedt auf der Günne*,[a]

[a,b] Inorganic Materials Chemistry, University of Siegen, Adolf-Reichwein-Str. 2, 57076 Siegen, Germany

*gunnej@chemie.uni-siegen.de

Supporting Information

1. Lattice parameters

Based on the Rietveld refinement results of the $La_{1-x}Nd_xPO_4$ series which were obtained by co-precipitation (Fig. **S1**), all the lattice parameters show linear correlation with doping level *x.*

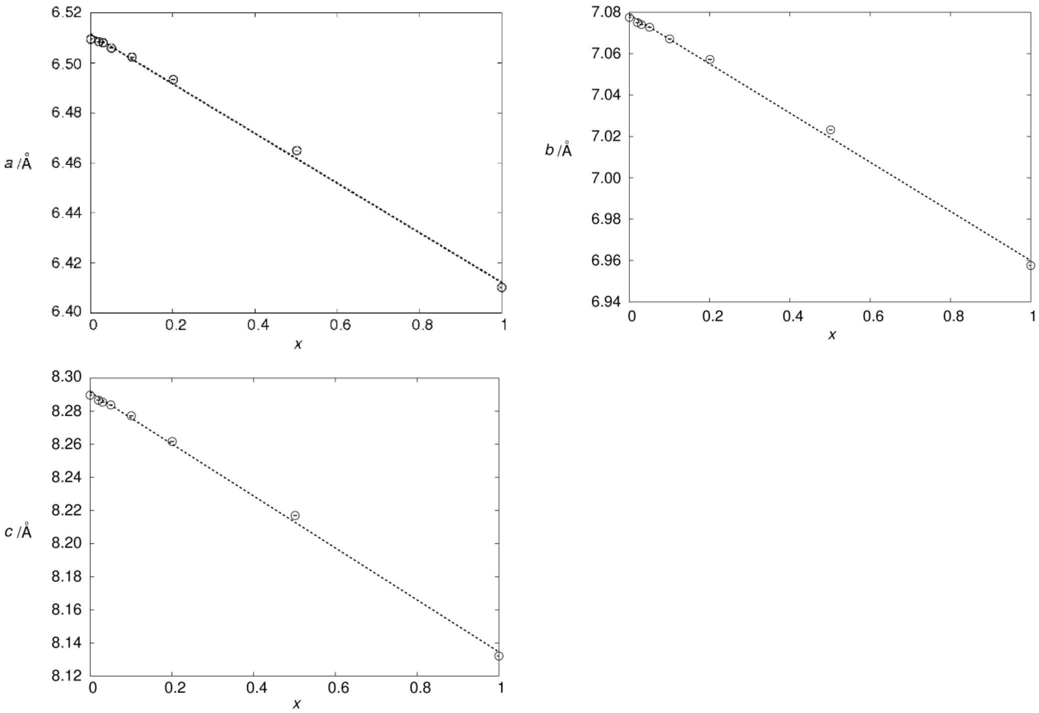

Figure S1. Lattice parameters as a function of the substitution degree x in the co-precipitated and 1000 °C sintered $La_{1-x}Nd_xPO_4$, as determined by Rietveld refinement based on X-ray powder diffraction data. The dotted lines represent linear fits resulting in a/Å = *6.5114* – 0.*0992*·x*,* b/Å = 7.0788 – 0.1189·x and c/Å = 8.2916 – 0.1570·x, respectively.

The Rietveld refinements have been performed also on the $La_{1-x}Dy_xPO_4$ (Fig. S2), $La_{1-x}Ho_xPO_4$ (Fig. S3), and $La_{1-x}Yb_xPO_4$ (Fig. S4) sample series, which were obtained by the co-precipitation method. The refinement results all show linear correlation with *x*. Note that the synthesized doping *x* ranges were different for different series, and at small doping level *x*, refinement data scattering is larger than at higher *x*.

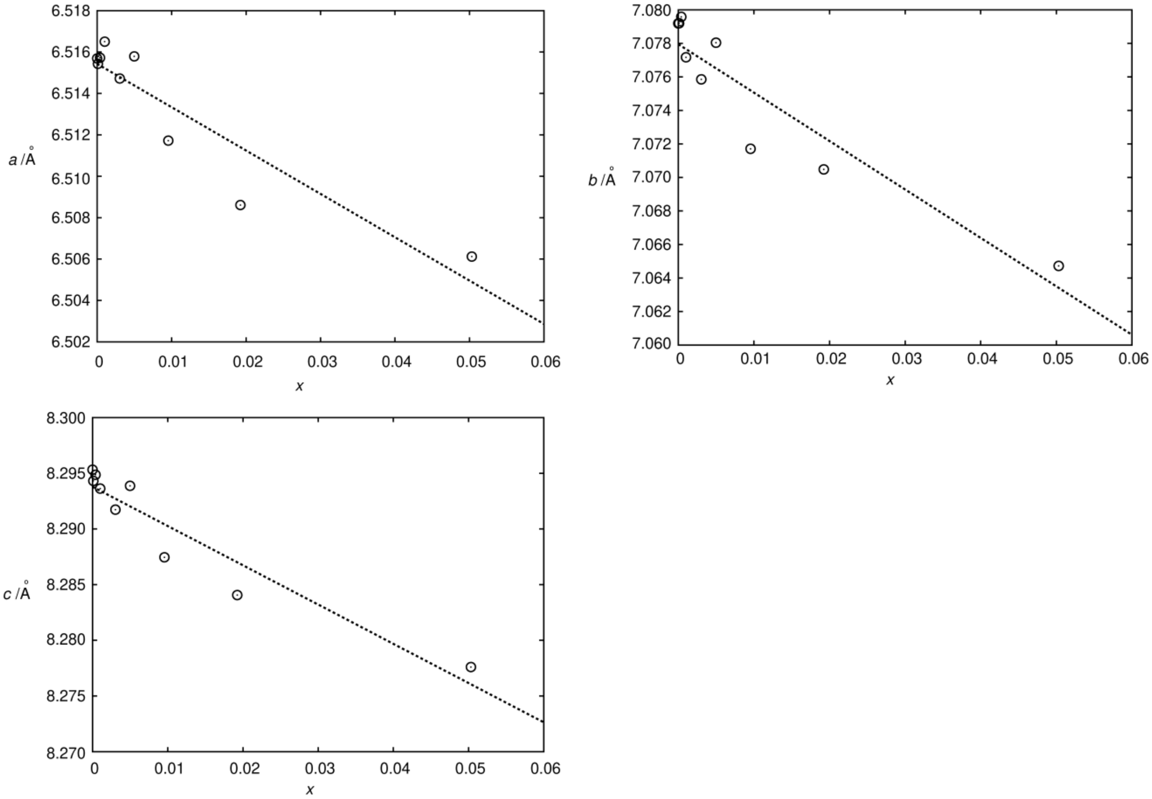

Figure S2. Lattice parameters as a function of the substitution degree *x* in the co-precipitated and 1000 °C sintered $La_{1-x}Dy_xPO_4$, as determined by Rietveld refinement based on X-ray powder diffraction data. The dotted lines represent linear fits resulting in a/Å = 6.5154 – 0.2094·x, b/Å = 7.0780 – 0.2890·x and c/Å = 8.2938 – 0.3525·x, respectively.

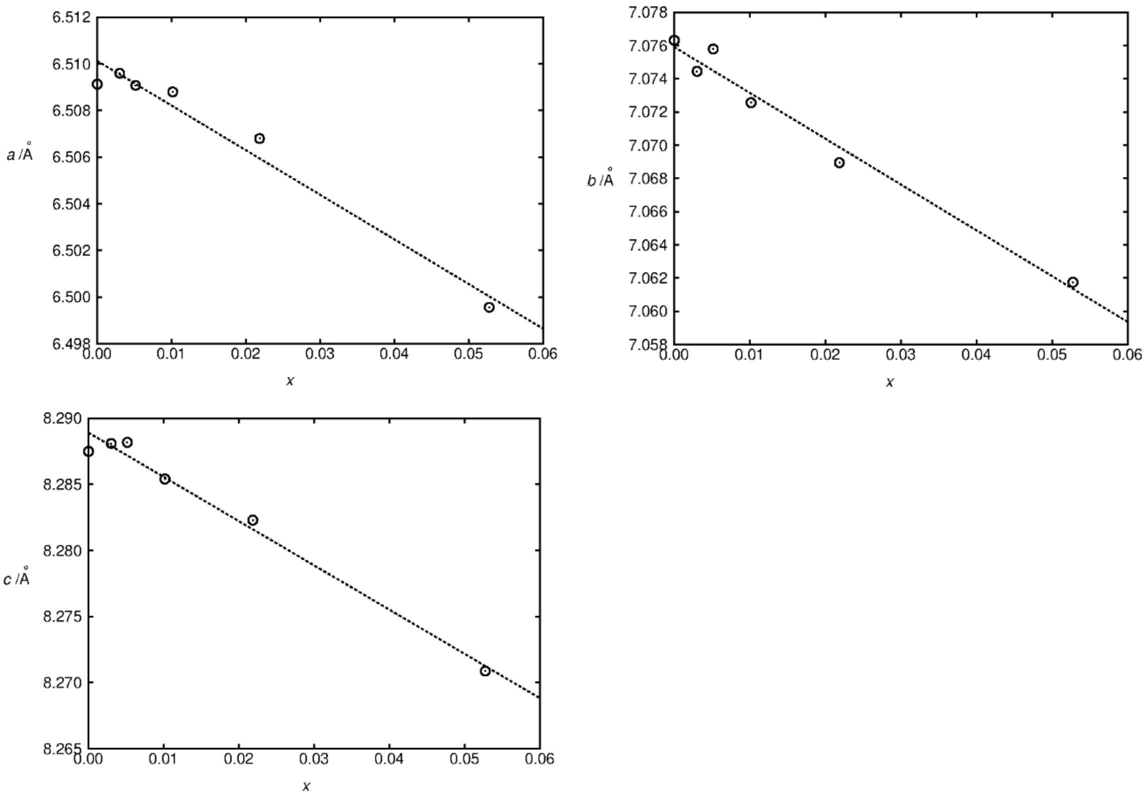

Figure S3. Lattice parameters as a function of the substitution degree *x* in the co-precipitated and 1000 °C sintered $La_{1-x}Ho_xPO_4$, as determined by Rietveld refinement based on X-ray powder diffraction data. The dotted lines represent linear fits resulting in *a*/Å = 6.5101 – 0.1914·*x*, *b*/Å = 7.0759 – 0.2761·*x* and *c*/Å = 8.2889 – 0.3349·*x*, respectively.

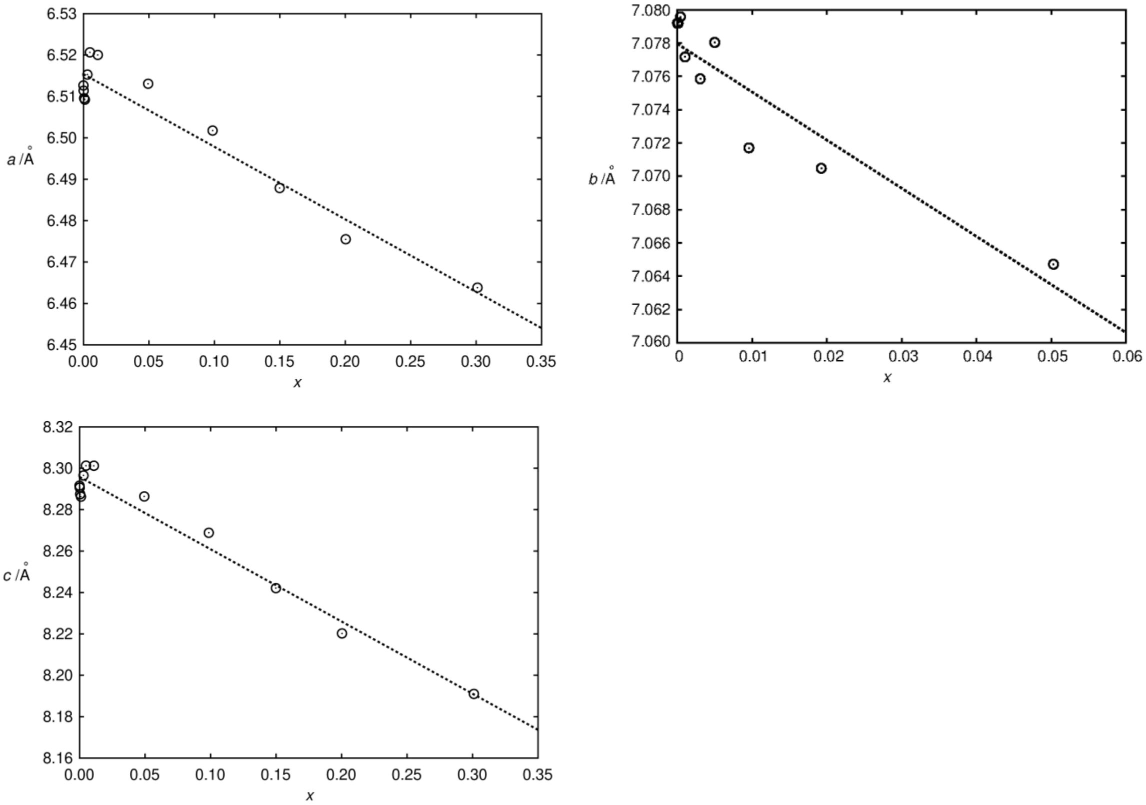

Figure S4. Lattice parameters as a function of the substitution degree *x* in the co-precipitated and 1000 °C sintered La$_{1-x}$Yb$_x$PO$_4$, as determined by Rietveld refinement based on X-ray powder diffraction data. The dotted lines represent linear fits resulting in a/Å = 6.5154 – 0.1753·x, b/Å = 7.0807 – 0.2994·x and c/Å = 8.2958 – 0.3492·x, respectively.

2. XRD diffractograms

For the 1000°C sintered solid state samples, at high doping concentration ($x \geq 0.2$), phase separation of $LnPO_4$ and $LaPO_4$ becomes evident from diffractograms. Nd doped $LaPO_4$ samples aimed at $x = 0.5$ were shown as examples. The XRD pattern of the solid state sample (Fig. S5 left) shown phase separation of $LaPO_4$ and $NdPO_4$. No phase separation was observed for corresponding co-precipitated sample (Fig. S5 right).

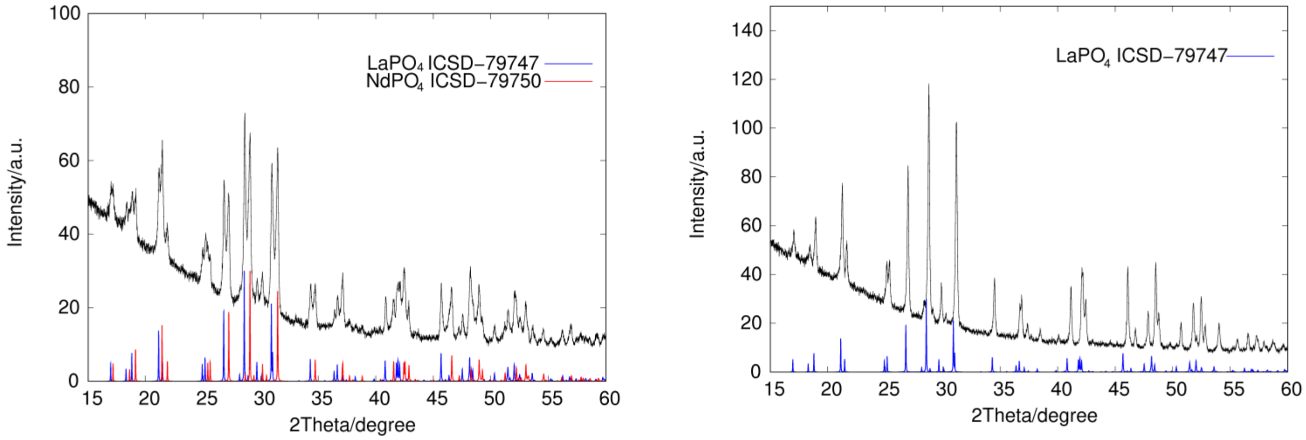

Figure S5. The powder XRD pattern of Nd doped $LaPO_4$ samples aimed at x=0.5, compared with literature data (ICSD-79747[1] for $LaPO_4$ and 79750[1] for $NdPO_4$). Left: solid state method synthesized, right: co-precipitation method synthesized sample.

3. NMR visibility curves

To establish the scale on which homogeneity is studied by NMR, the ^{31}P MAS NMR spectra were first obtained for the doped sample series $La_{1-x}Ln_xPO_4$ (Ln = Nd, Gd, Ho, Er, Tm, Yb). Only one signal was observed (Fig. S6) and as the doping level x increases, the peak area gradually decreases as shown in the ^{31}P MAS NMR spectra stack plots (Fig. S7-S12). Such data sets of the peak area of the homogeneous compounds $La_{1-x}Ln_xPO_4$ (Ln = Nd, Gd, Ho, Er, Tm, Yb) were fitted with the visibility function[2] $f(x) = \exp(-ar_0^3x)$ for the wipe-out radii r_0 of blind spheres. The $f(x)$ plots were shown next to the corresponding NMR stack plots in the Fig. S7-S12. NMR data from samples which were obtained from the solid state method were also compared along (Fig. S7-S12), and the deviation from the NMR visibility function indicates heterogeneity. For all mentioned Ln^{3+} dopants, homogeneously and heterogeneously doped samples can be distinguished. Therefore such method based on the

NMR visibility function serves as a nice tool for the evaluation of "NMR homogeneity". Based on the radii r_0 of the blind spheres,[3] it may be concluded that "NMR homogeneity" relies on a length scale of about 1 nm, and the co-precipitated samples (annealed at 1000 °C) are more homogeneously doped on nm scale.

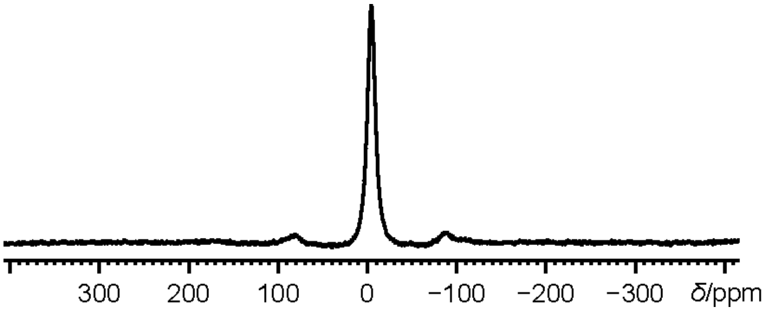

Figure S6. The ^{31}P MAS NMR full spectrum (spectrum width 100 kHz) of $La_{0.995}Dy_{0.005}PO_4$ obtained by co-precipitation method, which is shown as one example to demonstrate typical spectra of Ln doped $LaPO_4$ series. Only one P signal with its spinning side band were observed.

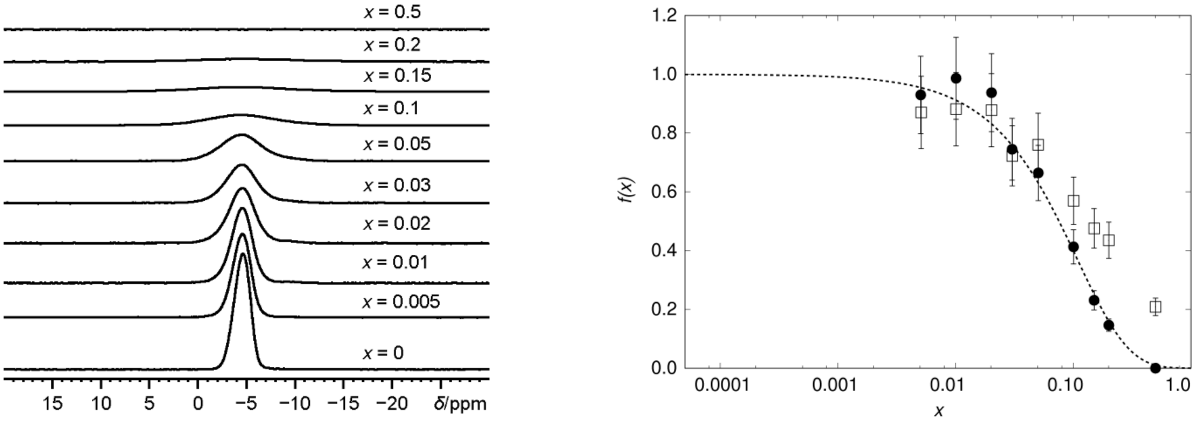

Figure S7. The stack plot of ^{31}P NMR spectra for $La_{1-x}Nd_xPO_4$ obtained by co-precipitation method (left), and the normalized NMR visibility $f(x)$ as a function of doping concentration x (right), for $La_{1-x}Nd_xPO_4$ obtained by co-precipitation method (circles) and solid state method (squares), both sintered at 1000 °C. The dashed line features the best fit of $f(x) = \exp(-ar^3x)$ with $a = 0.055/Å^3$ and $r_0 = 5.5$ Å to the points of samples from the co-precipitation method.

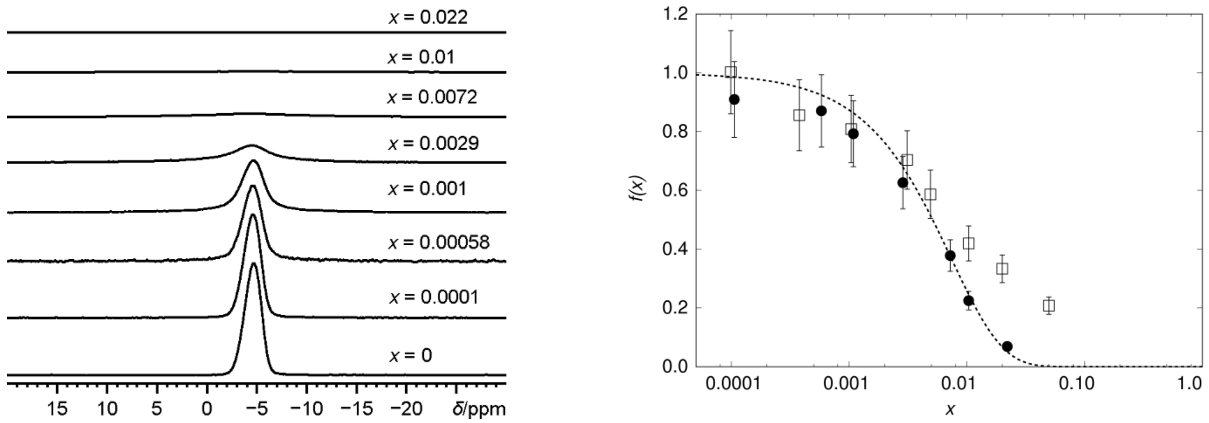

Figure S8. The stack plot of ^{31}P NMR spectra for $La_{1-x}Gd_xPO_4$ obtained by co-precipitation method (left), and the normalized NMR visibility $f(x)$ as a function of doping concentration x (right), for $La_{1-x}Gd_xPO_4$ obtained by co-precipitation method (circles) and solid state method (squares), both sintered at 1000 °C. The dashed line features the best fit of $f(x) = \exp(-ar^3x)$ with a = 0.055/Å^3 and r_0 = 13.5 Å to the points of samples from the co-precipitation method.

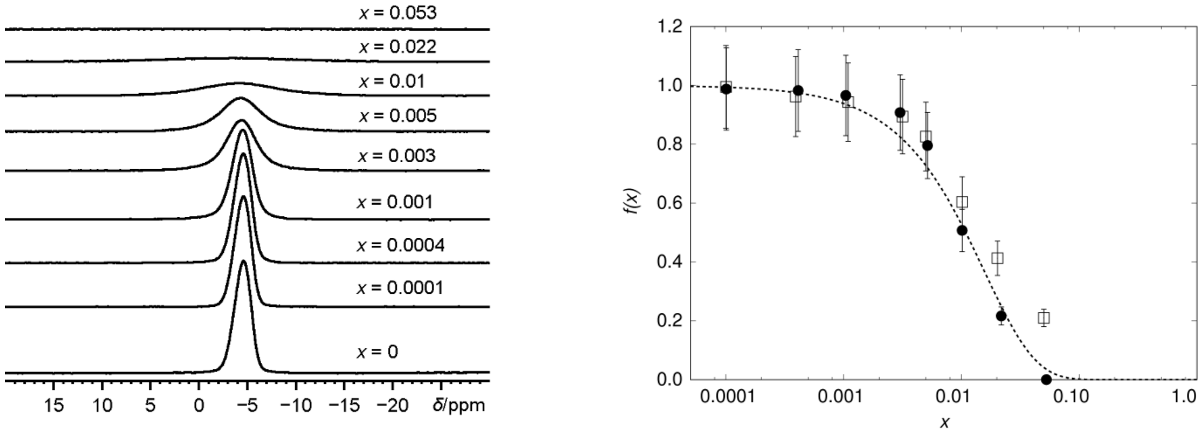

Figure S9. The stack plot of ^{31}P NMR spectra for $La_{1-x}Ho_xPO_4$ obtained by co-precipitation method (left) and the normalized NMR visibility $f(x)$ as a function of doping concentration x (right), for $La_{1-x}Ho_xPO_4$ obtained by co-precipitation method (circles) and solid state method (squares), both sintered at 1000 °C. The dashed line features the best fit of $f(x) = \exp(-ar^3x)$ with a = 0.055/Å^3 and r_0 = 10.5 Å to the points of samples from the co-precipitation method.

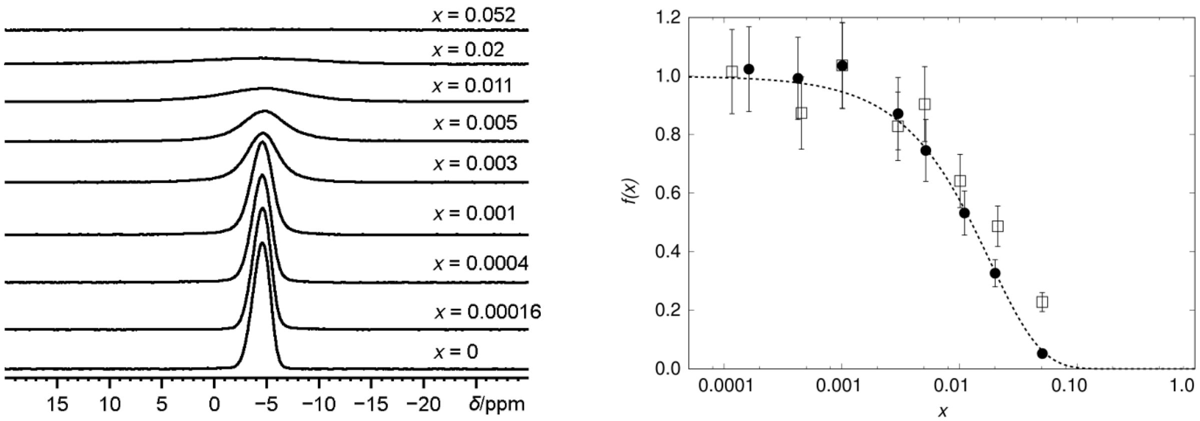

Figure S10. The stack plot of ^{31}P NMR spectra for $La_{1-x}Er_xPO_4$ obtained by co-precipitation method (left) and the normalized NMR visibility $f(x)$ as a function of doping concentration x (right), for $La_{1-x}Er_xPO_4$ obtained by co-precipitation method (circles) and solid state method (squares), both sintered at 1000 °C. The dashed line features the best fit of $f(x) = \exp(-ar^3x)$ with $a = 0.055/Å^3$ and $r_0 = 10$ Å to the points of samples from the co-precipitation method.

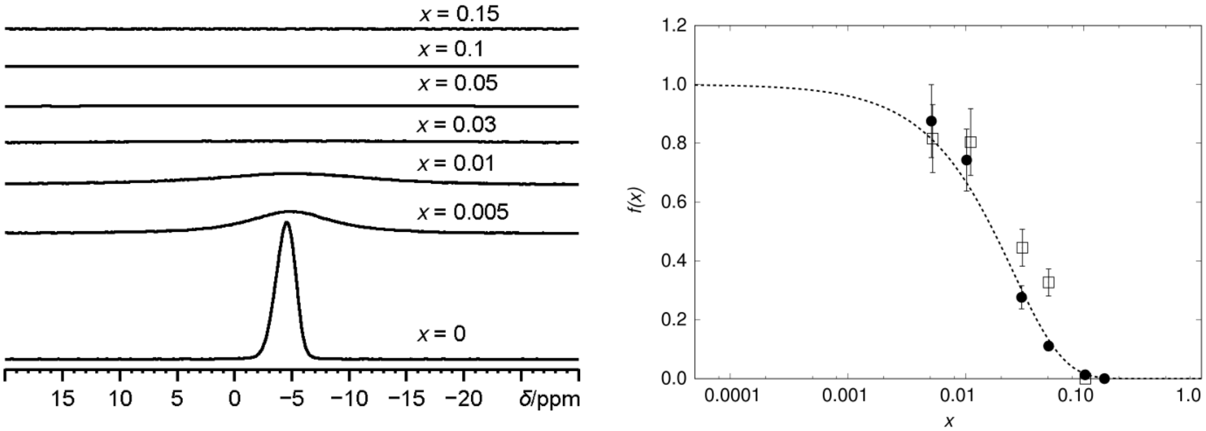

Figure S11. The stack plot of ^{31}P NMR spectra for $La_{1-x}Tm_xPO_4$ obtained by co-precipitation method (left) and the normalized NMR visibility $f(x)$ as a function of doping concentration x (right), for $La_{1-x}Tm_xPO_4$ obtained by co-precipitation method (circles) and solid state method (squares), both sintered at 1000 °C. The dashed line features the best fit of $f(x) = \exp(-ar^3x)$ with $a = 0.055/Å^3$ and $r_0 = 9$ Å to the points of samples from the co-precipitation method.

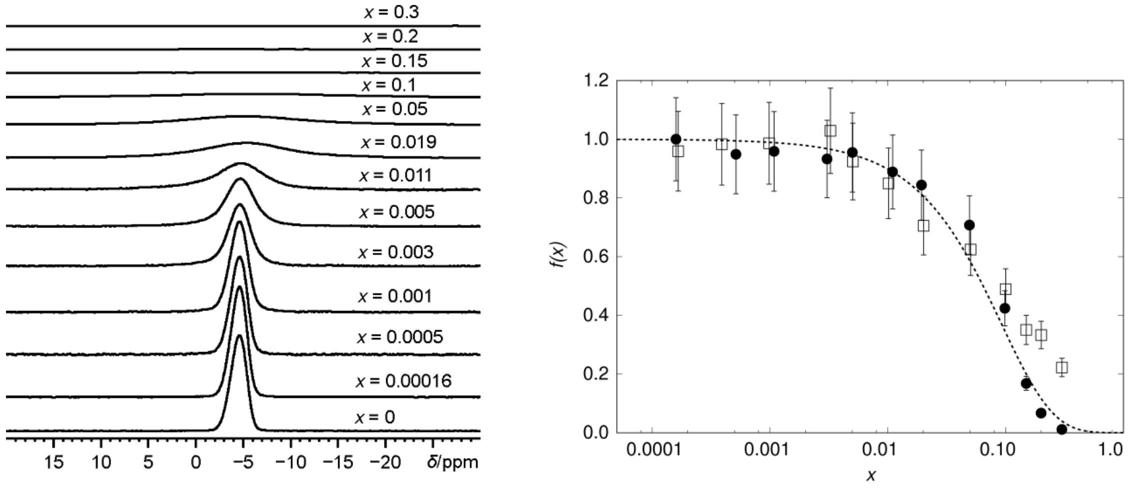

Figure S12. The stack plot of ^{31}P NMR spectra for $La_{1-x}Yb_xPO_4$ obtained by co-precipitation method (left) and the normalized NMR visibility $f(x)$ as a function of doping concentration x (right), for $La_{1-x}Yb_xPO_4$ obtained by co-precipitation method (circles) and solid state method (squares), both sintered at 1000 °C. The dashed line features the best fit of $f(x) = \exp(-ar^3x)$ with a = 0.055/Å^3 and r_0 = 5.8 Å to the points of samples from the co-precipitation method.

4. Lifetime of $La_{1-x}Dy_xPO_4$ doping series

The fluorescence lifetime of the $La_{1-x}Dy_xPO_4$ doping series has been obtained from the multi-exponential (equation **Fehler! Verweisquelle konnte nicht gefunden werden.** and 2) fitting of the intensity decay curve (Fig. S13-S19). I_{offset} was due to the dark counts and the corresponding parameter $c_{\text{offset}} = I_{\text{offset}}/I_0$ is also fitted (Table S1 and S2). I_0 is the initial intensity, which was set to be the intensity of the first data point in the decay curve. τ_1, τ_2 and τ_3 are the lifetime and a_1, a_2 and a_3 are the fitted weight fractions, respectively. In particular, a_1 is the mono-exponentiality factor.

The results shown in Tables S1 and S2 are the results of a single unconstrained non-linear least square fit of all slifetime curves with a self-written tcl-script including an error analysis based on a variance analysis. The fit model used different a_1, a_2, a_3 and I_{offset} / I_0 parameters for the different lifetime curves but assumed the lifetime values $\tau_1,\ \tau_2$ and τ_3 to be the same for both sample series. The fit converged consistently to the same minimal value independent of small changes in the starting values.

The values in Table S1 and S2 were used to produce Figure 6 in the manuscript.

Table S1. The fitting parameters including weight fractions a_1, a_2, a_3 and intensity offset c_{offset} for the lifetime measurements of samples obtained from co-precipitation method. The lifetime values are $\tau_1 = 1.099 \pm 0.003$ ms, $\tau_2 = 0.583 \pm 0.007$ ms, $\tau_3 = 0.099 \pm 0.002$ ms.

x	a_1	a_2	a_3	c_{offset}
0.0004	0.921±0.009	0.034±0.008	0.045±0.005	0.0049±0.0002
0.001	0.920±0.009	0.080±0.008	0.000±0.005	0.0008±0.0002
0.003	0.866±0.008	0.128±0.007	0.006±0.005	0.0000±0.0002
0.005	0.816±0.007	0.071±0.006	0.114±0.004	0.0006±0.0002
0.01	0.549±0.006	0.428±0.006	0.023±0.005	0.0019±0.0002
0.02	0.451±0.007	0.511±0.008	0.038±0.007	0.0018±0.0003
0.05	0.000±0.010	0.660±0.008	0.340±0.005	0.0059±0.0006

Table S2. The fitting parameters including weight fractions a_1, a_2, a_3 and intensity offset I_{offset} for the lifetime measurements of samples obtained from solid state method.

x	a_1	a_2	a_3	c_{offset}
0.0004	0.898±0.009	0.000±0.008	0.102±0.005	0.0055±0.0002
0.001	0.901±0.008	0.019±0.008	0.080±0.005	0.0022±0.0002
0.003	0.773±0.006	0.101±0.006	0.126±0.004	0.0011±0.0002
0.005	0.684±0.006	0.169±0.005	0.147±0.004	0.0004±0.0002
0.01	0.508±0.007	0.421±0.007	0.072±0.007	0.0007±0.0003
0.02	0.337±0.007	0.573±0.008	0.091±0.007	0.0006±0.0003
0.05	0.116±0.007	0.529±0.006	0.355±0.005	0.0041±0.0005

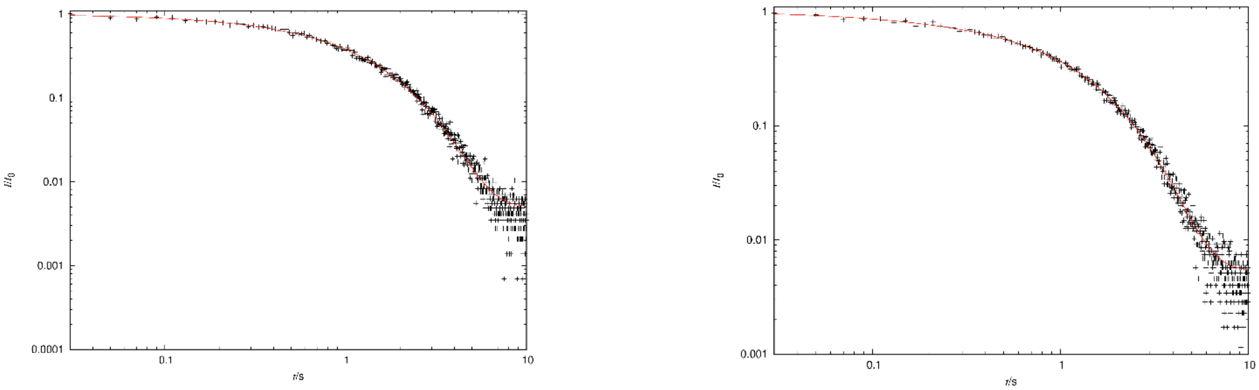

Figure S13. The decay curves from lifetime measurements of $La_{0.9996}Dy_{0.0004}PO_4$ samples, obtained by co-precipitation method with sintering temperature 1000°C (left) and solid state method 1000°C (right). The dashed lines represent the fitting function as equation 2 with fitting parameters in table S1 (left) and S2 (right), respectively. The measurements were recorded at emission wavelength λ_{em} = 477 nm and excitation wavelength λ_{ex} = 350 nm.

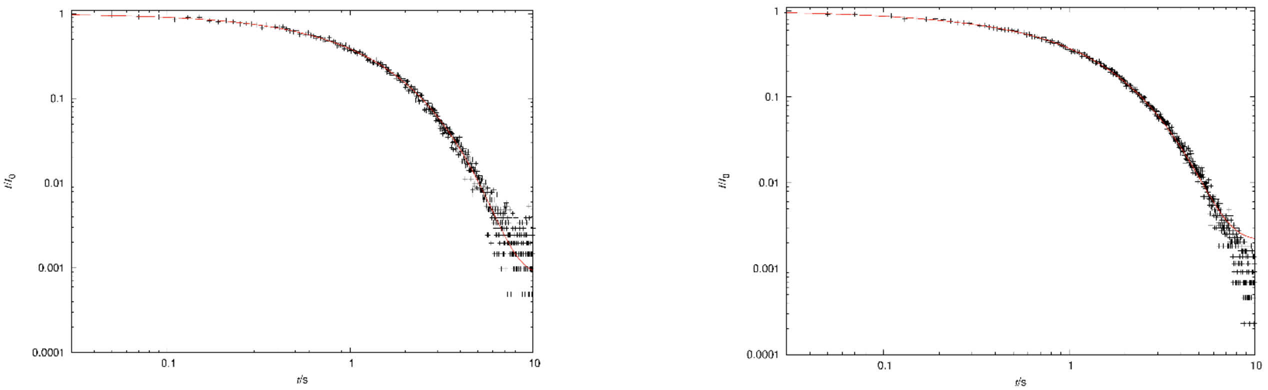

Figure S14. The decay curves from lifetime measurements of $La_{0.999}Dy_{0.001}PO_4$ samples, obtained by co-precipitation method with sintering temperature 1000°C (left) and solid state method 1000°C (right). The dashed lines represent the fitting function as equation 2 with fitting parameters in table S1 (left) and S2 (right), respectively. The measurements were recorded at emission wavelength λ_{em} = 477 nm and excitation wavelength λ_{ex} = 350 nm.

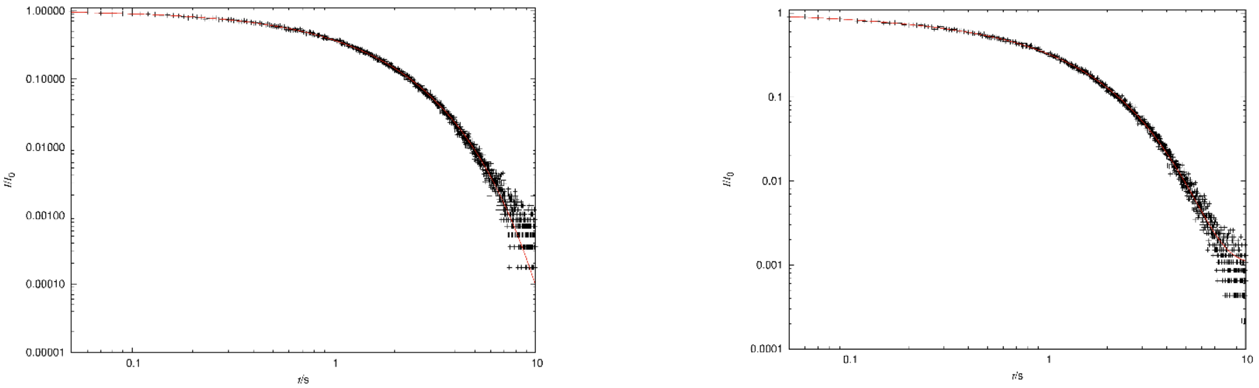

Figure S15. The decay curves from lifetime measurements of $La_{0.997}Dy_{0.003}PO_4$ samples, obtained by co-precipitation method with sintering temperature 1000°C (left) and solid state method 1000°C (right). The dashed lines represent the fitting function as equation 2 with fitting parameters in table S1 (left) and S2 (right), respectively. The measurements were recorded at emission wavelength λ_{em} = 477 nm and excitation wavelength λ_{ex} = 350 nm.

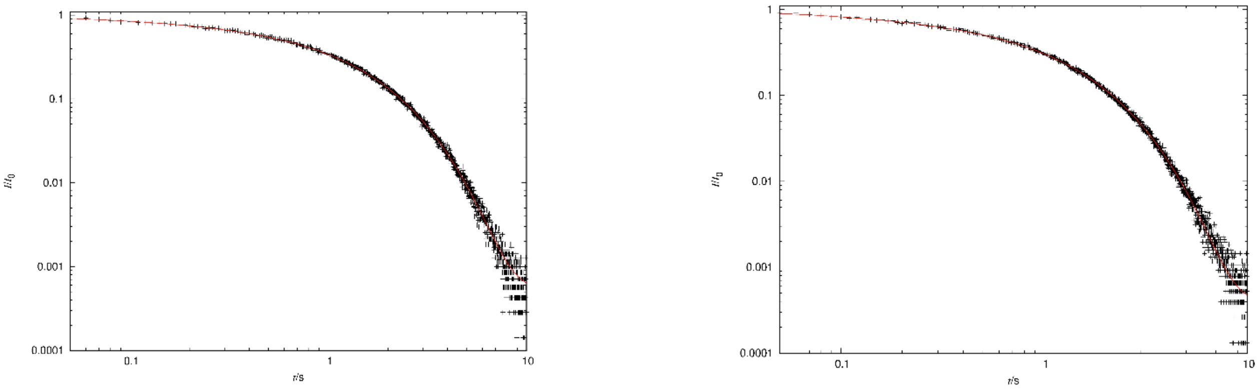

Figure S16. The decay curves from lifetime measurements of $La_{0.995}Dy_{0.005}PO_4$ samples, obtained by co-precipitation method with sintering temperature 1000°C (left) and solid state method 1000°C (right). The dashed lines represent the fitting function as equation 2 with fitting parameters in table S1 (left) and S2 (right), respectively. The measurements were recorded at emission wavelength λ_{em} = 477 nm and excitation wavelength λ_{ex} = 350 nm.

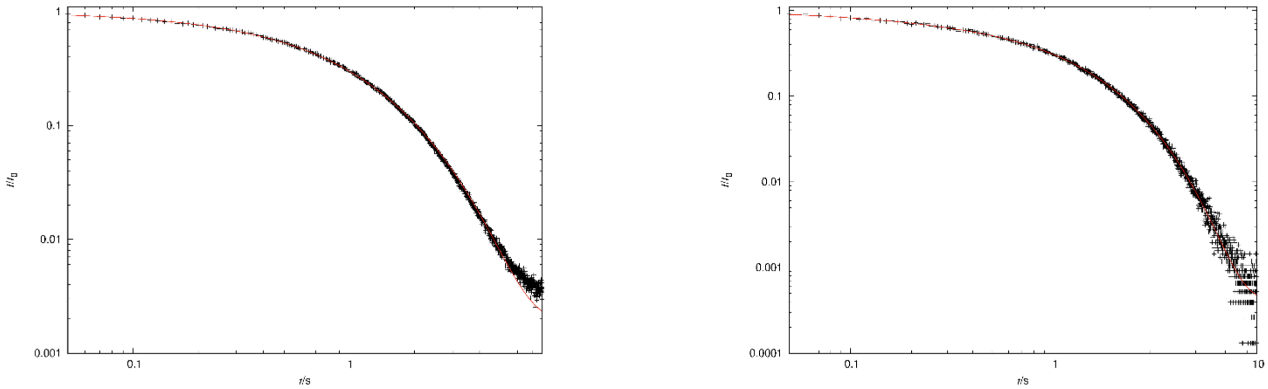

Figure S17. The decay curves from lifetime measurements of $La_{0.99}Dy_{0.01}PO_4$ samples, obtained by co-precipitation method with sintering temperature 1000°C (left) and solid state method 1000°C (right). The dashed lines represent the fitting function as equation 2 with fitting parameters in table S1 (left) and S2 (right), respectively. The measurements were recorded at emission wavelength λ_{em} = 477 nm and excitation wavelength λ_{ex} = 350 nm.

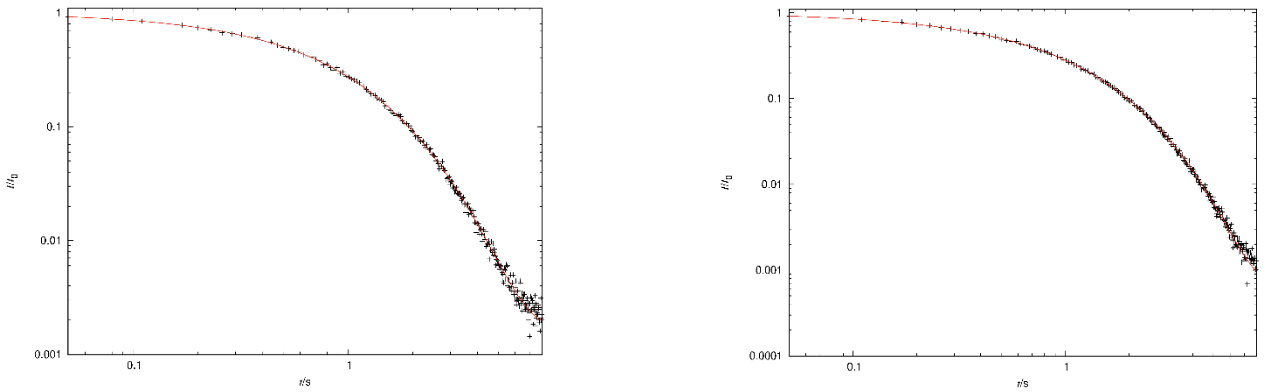

Figure S18. The decay curves from lifetime measurements of $La_{0.98}Dy_{0.02}PO_4$ samples, obtained by co-precipitation method with sintering temperature 1000°C (left) and solid state method 1000°C (right). The dashed lines represent the fitting function as equation 2 with fitting parameters in table S1 (left) and S2 (right), respectively. The measurements were recorded at emission wavelength λ_{em} = 477 nm and excitation wavelength λ_{ex} = 350 nm.

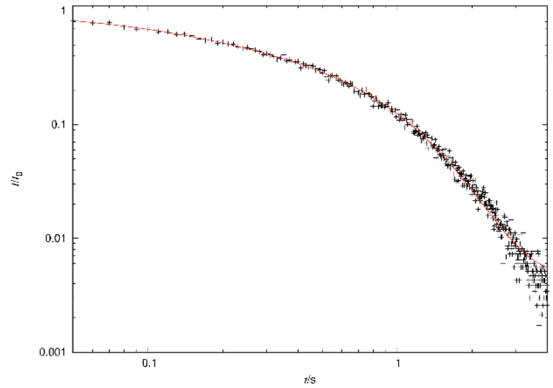

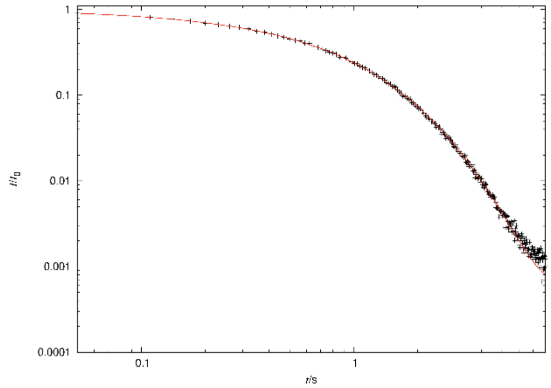

Figure S19. The decay curves from lifetime measurements of $La_{0.95}Dy_{0.05}PO_4$ samples, obtained by co-precipitation method with sintering temperature 1000°C (left) and solid state method 1000°C (right). The dashed lines represent the fitting function as equation 2 with fitting parameters in table S1 (left) and S2 (right), respectively. The measurements were recorded at emission wavelength λ_{em} = 477 nm and excitation wavelength λ_{ex} = 350 nm.

References

[1] Y. Ni, J. M. Hughes , A. N. Mariano, *Am. Mineral.*, **1995**, *80*, 21–26.

[2] W. Li, V. R. Celinski, J. Weber, N. Kunkel, H. Kohlmann, J. Schmedt auf der Günne, *Phys. Chem. Chem. Phys.* **2016**, *18*, 9752–9757.

[3] W. Li, Q. Zhang, J. J. Joos, P. F. Smet, J. Schmedt auf der Günne, *Phys. Chem. Chem. Phys.* **2019**, *21*, 10185–10194.
